# Adolescents’ Self and Bullying Victimisation: The Key Role of Gender

**DOI:** 10.3390/children12020217

**Published:** 2025-02-12

**Authors:** Matteo Perazzini, Danilo Bontempo, Marco Giancola, Simonetta D’Amico, Enrico Perilli

**Affiliations:** 1 Department of Life, Health, and Environmental Sciences, University of L’Aquila, 67100 L’Aquila, Italy; danilo.bontempo@guest.univaq.it (D.B.); enrico.perilli@univaq.it (E.P.); 2 Department of Biotechnological and Applied Clinical Sciences, University of L’Aquila, 67100 L’Aquila, Italy; marco.giancola@univaq.it (M.G.); simonetta.damico@univaq.it (S.D.)

**Keywords:** bullying, self, adolescence, gender, moderation

## Abstract

**Background/Objectives**: The primary factors influencing victimisation in bullying have recently become a topic of debate in the psychological literature. This research sought to explore the association between self-criticism (as captured in terms of inadequate self, hated self, and reassured self) and bullying victimisation, also addressing the moderating effects of gender. **Methods**: This study relies on a cross-sectional research design, enrolling a sample of 328 adolescents (M_age_ = 14.93; SD_age_ = 0.81; 162 females, 49.4%). Participants completed a short questionnaire about their age, gender, and family’s socio-economic status. They also answered questions from the Forms of Self-Criticising/Attacking and Self-Reassuring Scale and the Florence Bullying and Victimisation Scales. **Results**: Statistical analysis indicated gender moderated only the interplay between hated self and bullying victimisation. **Conclusions**: Through a multidimensional approach, these findings provided further insight into the primary factors of self to the phenomenon of bullying victimisation, also yielding further evidence about the key role of gender during a critical developmental stage, such as adolescence. Limitations, implications, and future research directions are discussed.

## 1. Introduction

Bullying is a complex social problem characterised by ongoing aggressive behaviours, including physical and verbal abuse, social exclusion, and tactics, such as intimidation and manipulation. It involves a noticeable power imbalance between the victim and the bully. This power disparity is crucial, as it enables the bully to assert control and dominance over the victim, who often finds it challenging to defend themself effectively [1,2,3,4,5]. This imbalance can take various forms, including physical force, social hierarchy, or psychological manipulation, serving as a defining trait that sets bullying apart from other forms of aggression [6,7].

Bullying extends beyond a simple one-on-one interaction between an aggressor and a victim: it manifests as a multifaceted social dynamic, encompassing various roles and participants. Beyond the aggressor and the victim, this landscape includes supporters, defenders of the victim, and bystanders [8]. Each of these roles plays a crucial part in shaping the bullying dynamics, either perpetuating harmful behaviours or helping to alleviate them within a social group. In particular, the followers engage directly in the bullying behaviour or tacitly endorse it by their mere presence and approval. Defenders advocate for the victim, intervening to prevent bullying or offering emotional support. In turn, bystanders often adopt a passive stance, choosing not to engage with either the aggressor or the victim, typically driven by the desire to avoid conflict or a lack of confidence in their ability to manage the situation [5]. This depicts the multifaceted nature of bullying as a social dynamic, where peer behaviours and attitudes play a crucial role in both the manifestation and continuation of bullying [9,10].

Research on bullying in Italy revealed alarming trends, particularly in central and southern regions. Findings indicate that the prevalence of bullying in Italy exceeds the levels observed in countries such as Norway, England, Spain, and Japan. In addition, research highlighted that bullying tends to decline as individuals grow older, with boys more frequently acknowledging their role in bullying behaviour [11]. In central Italy, previous research [12,13] revealed that over half of middle school students reported experiencing bullying within the previous three months, with nearly half identifying as victims.

Understanding the main psychological characteristics of victims is essential for the formulation of targeted interventions and preventive strategies. Scientific research identifies some psychological characteristics and gender differences that outline typical victim profiles [14]. Bullying victims fall into two main categories: passive victims and defiant victims [1]. Passive victims typically display traits of introversion, insecurity, and lack of assertiveness. They exhibit low self-confidence and self-esteem, making them vulnerable to aggression [15]. Often, passive victims struggle to react to bullying, preferring to avoid conflict or submit to aggressors, which perpetuates the bullying cycle. In contrast, defiant victims exhibit traits of both victims and aggressors. Defiant victims demonstrate higher levels of emotional dysregulation and social difficulties compared to peers who are solely aggressors or victims [16,17]. These individuals often display a poor psychosocial profile, including reactive aggression, anxiety, and depressive symptoms [18,19]. In cases of childhood abuse, victims who identify with aggressors exhibit both inward (self-injurious behaviour) and outward aggression (violent acts toward others) [20]. In school settings, defiant victims often engage in reciprocal aggressive relationships, acting as both perpetrators and victims of aggression [21]. Defiant victims frequently show hyperactivity and impulsivity, provoking negative reactions from peers with irritating or destructive behaviour [22]. Both passive and defiant victims often lack close relationships with peers or family that could shield them from bullying [23]. Studies showed that being a victim of bullying significantly increases the risk of emotion regulation issues, anxiety, depression, and post-traumatic stress symptoms, which weaken the overall quality of life and increase the likelihood of self-harm or suicidal ideation [24,25,26,27].

In light of the detrimental effects associated with being a victim of bullying, research aims to elucidate the primary psychological risk factors that expose individuals to victimisation. Among these, the concept of self-perception is gaining considerable traction within the field of psychological research. Gilbert’s model [28] defines self-perception through three main dimensions: inadequate self (or self-criticism), hated self (or self-attacking), and reassured self (or self-reassuring). Regarding inadequate self, Freud [29] formalised this experience by suggesting that depression-linked self-devaluation and criticism arise from Superego attacks on the Ego and efforts to protect a needed person from anger. Overall, inadequate self involves feelings of inadequacy, self-doubt, and a tendency to dwell on mistakes, reflecting a general form of self-criticism where individuals feel that they are not good enough [30,31]. In turn, hated self represents a more severe form, involving self-hatred, self-disgust, and a desire to harm oneself. It is associated with more intense negative emotions and a punitive attitude toward oneself [28,31]. In contrast, reassured self reflects the ability to be kind and supportive to oneself, especially during failure or distress. It indicates a compassionate and soothing self-to-self relationship [30,31].

The association between self-perception and victimisation still remains underexplored. However, some studies indicated a positive correlation between inadequate self, hated self, and bullying victimisation [32,33]. Furthermore, aspects of inadequate self, such as low self-esteem, represent critical factors in explaining bullying victimisation. Indeed, victims of bullying often exhibit lower self-esteem, contributing to a cycle of victimisation and further psychological distress [32]. Victims frequently show a tendency to self-blame, positively correlated with depression. This tendency appears more pronounced in relational bullying compared to verbal and physical bullying. Additionally, individuals who are both perpetrators and victims tend to show higher self-blame tendencies compared to victims alone [34,35,36]. As for self-reassuring, Duarte and colleagues [37] highlighted the importance of assessing the quality of adolescents’ interpersonal experiences and their association with self-assurance skills.

### The Moderating Role of Gender

Bullying victimisation is a phenomenon that impacts both males and females, although the modalities of expression and prevalence may differ considerably between the two genders. Boys are more likely to experience direct physical or verbal bullying, while girls are more prone to indirect forms, such as social exclusion and gossip [27,38,39]. Although boys tend to be more involved as both aggressors and victims of physical bullying, girls are often engaged in relational bullying, which is less visible but equally harmful, affecting victims’ social relationships and self-esteem [40]. These patterns reflect cultural and social pressures, including conformity to gender roles, which enhance specific bullying behaviours [38,41]. Indeed, gender differences in bullying can vary with age and context. During preadolescence, boys tend to experience higher rates of bullying, but this gap narrows in adolescence, particularly with the rise in online bullying [42,43]. While boys are more likely to be victims of physical aggression, girls report higher rates of cyberbullying, highlighting distinct modalities of victimisation [44,45].

Interestingly, gender differences can also be related to self-perception. Indeed, some studies showed that gender differences in inadequate and hated self emerge clearly, with men and women tending to experience this condition in distinct ways due to social norms and emotion regulation as well as biological dynamics [46].

As for social norms and emotion regulation, women are subject to strong social pressure related to beauty standards and physical appearance. This often leads to increased body dissatisfaction and heightened inadequate self [47]. The objectification of the female body, promoted by the media and popular culture, leads many women to evaluate themselves through an ‘outside eye’, causing feelings of shame and inadequacy [47]. Men, on the other hand, are pressured to adhere to ideals of strength, power, and success. Failure to meet these socially imposed standards can generate feelings of failure and self-hatred, often linked to a sense of inadequacy in relation to the traditional role of a ‘provider’ and dominant figure [48]. These dynamics are intensified in contexts where men are discouraged from showing vulnerability, leading them to internalise failure without the possibility of emotional expression. This often leads to increased body dissatisfaction and heightened inadequate self and self-hatred differently between genders [49,50].

As for biological dynamics, hormonal changes during critical periods such as puberty, pregnancy, and menopause can increase vulnerability to negative emotions and self-hatred [51]. These hormonal changes can interact with social and psychological factors, creating a complex web of influences that fuel self-hatred. In men, lower levels of emotional awareness and a tendency to develop alexithymia—difficulty identifying and describing emotions—can hinder the recognition and management of feelings of self-hatred [52]. This difficulty can make it more difficult for men to deal with negative feelings, perpetuating cycles of self-criticism and dissatisfaction.

Based on this mechanism, this research advanced that girls who report experiencing less inadequate self and hated self tend to be less victimised. Indeed, previous research supports the widespread hypothesis of being female as a protective factor as women vary more than men in their use of emotional strategies [53]. Girls tend to develop greater emotional awareness and emotional regulation skills than boys, which helps them better manage feelings of inadequacy as well as reducing the likelihood of becoming targets for bullies [53,54]. Therefore, the first two hypotheses are as follows:

**H1.**  *Gender moderates the association between inadequate self and victimisation, weakening this association.*

**H2.**  *Gender moderates the association between hated self and victimisation, weakening this association.*

As for the potential moderating effect of gender in the association between reassuring self and victimisation, no clear evidence can be useful to explain the role of being female. However, it is reasonable that the female gender may moderate the interplay between self-reassuring and bullying victimisation due to girls being more likely to employ emotion regulation strategies (e.g., cognitive emotional regulation) based on social support and self-reflection compared to boys [55,56]. This may enhance the effectiveness of self-reassuring in mitigating the emotional impact of bullying, serving as a protective factor against negative situations, such as being victimised by bullies. Therefore, the hypothesis is as follows:

**H3.**  *Gender (being female) moderates the association between reassuring self and bullying victimisation, buffering this association.*

## 2. Materials and Methods

### 2.1. Participants and Procedure

The research sample consisted of 328 adolescents (M_age_ = 14.93; SD_age_ = 0.81; 162 females, 49.4%). Participants were recruited through schools in central Italy. Before the survey began, participants and their parents received clear information about the study’s purpose through an informed consent page, ensuring transparency and voluntary participation. The survey had two sections: the first gathered socio-demographic information like age and gender, and the second included self-report questionnaires, such as the Forms of Self-Criticising/Attacking and Self-Reassuring Scale and the Florence Bullying–Victimisation Scales. The participation in the study was voluntary with no rewards provided. Complete anonymity was guaranteed.

### 2.2. Measures

The Forms of Self-Criticising/Attacking and Self-Reassuring Scale (FSCRS) [28] consists of 22 items on a five-point Likert scale, ranging from 0 (not at all like me) to 4 (extremely like me), assessing the way people “treat themselves” when things go wrong. The FSCRS relies on three self-dimensions: inadequate self, which assesses feelings of inadequacy and a sense of irritation and frustration toward the self (e.g., “When things go wrong for me I am easily disappointed with myself”); hated self, which assesses a more extreme form of self-criticism, characterised by feelings of self-repugnance and the desire to hurt the self in response to failures and setbacks (e.g., “I have a sense of disgust with myself”); and reassured self, which indicates the ability to soothe the self with kindness and compassion when facing faults and failures (e.g., “I am gentle and supportive with myself”). As an Italian version is missing, the original 22 items of the FSCRS were translated and backtranslated. In this study, internal consistency for inadequate self, hated self, and reassuring self was Cronbach’s α = 0.81, 0.82, and 0.75, respectively.

The Florence Bullying–Victimisation Scales (FBVSs) [57] consist of 20 items asking how often in the last couple of months the adolescents had experienced certain behaviours, either as perpetrators or victims (e.g., “I threatened someone”; “I was threatened”). A definition of bullying introduced the scale. Each item was evaluated along a 5-point scale from “never” to “several times a week”. The two subscales consist of 10 items each. Given the aim of this research, only the subscale for victimisation was employed in this study. The internal consistency for victimisation was Cronbach’s α = 0.80.

### 2.3. Statistical Analysis

All statistical analyses were performed using SPSS Statistics version 24 for Windows (IBM Corporation, Armonk, NY, USA). To verify the relationships among the study variables, bivariate correlations were computed, while to test the moderating effect of gender in the association between self and bullying victimisation, the PROCESS macro for SPSS was used [58]. The significance of the moderating effect was analysed using 5000 resamples of bootstrapped estimates with 95% bias-corrected confidence intervals—CIs [59,60,61,62]. The 95% CIs must not cross zero to satisfy the criteria of moderation [63,64,65]. All significance was set to *p* < 0.05.

## 3. Results

A data distribution test revealed that all study variables were not normally distributed, while the z-test with ±4.0 z-scores as a cutoff [66,67,68] indicated no univariate outliers. As shown in Table 1, Spearman’s correlation indicated that bullying victimisation was positively associated with inadequate self (*r* = 0.26, *p* < 0.01) and hated self (*r* = 0.26, *p* < 0.01), and negatively with reassuring self (*r* = −0.12, *p* < 0.05). In addition, gender was negatively associated with inadequate self (*r* = −0.17, *p* < 0.01), and hated self (*r* = −0.11, *p* < 0.05), and positively with reassuring self (*r* = 0.24, *p* < 0.01).

Additionally, the moderation analysis was computed, entering one by one inadequate self, hated self, and reassuring self as the independent variables, bullying victimisation as the dependent variable, and gender as the moderator.

**Figure 1:**
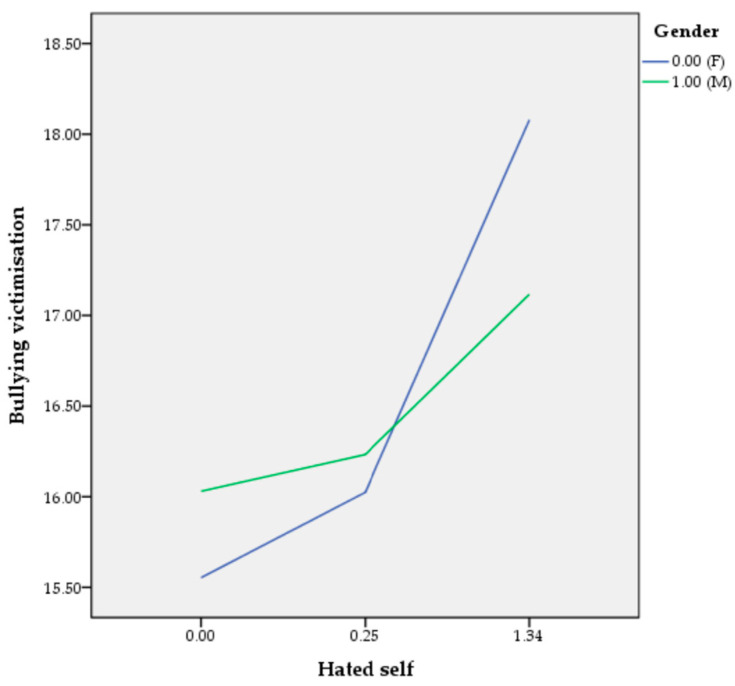
The moderation effect of gender in the association between hated self and bullying victimisation.

By entering inadequate self as the independent variable, results showed no interaction effect between inadequate self and gender (B = −0.41, SE = 0.44, t = −0.93; 95% CIs [−1.2717, 0.4544]). In addition, by using hated self as the independent variable, we found a significant interaction between hated self and bullying victimisation (B = −1.07, SE = 0.48, t = −2.24; 95% CIs [−2.0109, −0.1293]): hated self predicts bullying victimisation for girls (B = 1.89, SE = 0.35, t = 5.35; 95% CIs [1.1919, 2.5781]) and boys (B = 0.81, SE = 0.33, t = 2.48; 95% CIs [0.1682, 1.4531]) (Figure 1). The *R*^2^ for the entire model was 0.10 (F4,323 = 8.86, *p* < 0.001). Finally, with reassuring self as the independent variable, the analysis indicated no interaction effect between reassuring self and gender (B = −0.21, SE = 0.52, t = −0.41; 95% CIs [−1.2271, 0.8004]).

## 4. Discussion

This research sought to examine the association between self (as captured in terms of inadequate self, hated self, and reassuring self) and bullying victimisation, addressing the moderating effects of gender.

The moderation analysis revealed that the female gender had a stronger moderating effect than the male gender only in the association between hated self and bullying victimisation. These results showed that girls with hated self (i.e., a negative self-perception, characterised by self-hatred, self-disgust, and a desire to harm oneself) are less likely to experience bullying victimisation than boys. Given that, findings confirmed H2 and rejected H1 and H3.

Although some studies suggested that girls with negative self-perception are more vulnerable to bullying due to relational and emotional sensitivity [69,70], our findings align with other research suggesting that girls are more able to face risk behaviours [71]. One possible explanation can rely on emotion regulation strategies that protect them from potentially harmful behaviours [71,72,73]. Indeed, girls use acceptance and problem-solving strategies more often than boys, which helps them manage negative emotions better. These strategies are linked to fewer depressive symptoms and may protect them from bullying victimisation. They use cognitive reappraisal more effectively, which means that they change their thoughts about a situation to reduce its emotional impact [73,74]. Girls generally receive more social support from peers and parents, which helps them cope with negative emotions [75].

Another explanation could be provided considering gender differences in emotional intelligence. Quintana-Orts and colleagues [76] suggested that being female serves as a protective factor against hated self and bullying victimisation, mainly due to elevated emotional intelligence. Indeed, women with emotional intelligence are more able to cope with unpleasant emotions. Women with emotional intelligence are better able to handle unpleasant emotions, and the mediating effect of emotional intelligence on reducing the risk of bullying victimisation is stronger in girls than in boys [76]. Furthermore, women with high social intelligence tend to show better emotional management and pro-social behaviours, which may contribute to lower rates of victimisation [77], supporting our hypothesis and a highly protective factor. Emotional intelligence is linked to a decrease in victimisation in bullying contexts because it acts as a buffer by improving emotional regulation and understanding, which reduces the likelihood of being bullied. High emotional intelligence is linked to lower bullying victimisation and improved psychological adjustment, indicating that it helps reduce the harmful effects of bullying [76,78,79]. Furthermore, high emotional intelligence reduces bullying victimisation and improves psychological well-being as a protective factor against bullying’s negative effects [79,80].

The findings provided by this research offer both theoretical and practical implications for a more nuanced understanding of bullying victimisation. From a theoretical point of view, this study advances the literature by elucidating the critical role of gender as a moderating factor in the relationship between self-perception and bullying victimisation. Specifically, the moderating effect of gender was found in the association between hated self and victimisation, highlighting the contribution of gendered patterns that may guide future research. From a practical point of view, the findings provided by this research underscore the need for tailored interventions and specific guidelines that could reduce bullying victimisation. Programmes designed to foster emotional strategies (cognitive reappraisal) and emotional intelligence tailored to gender-specific needs could be helpful to mitigate the detrimental effect of hated self and, consequently, reduce the risk of victimisation. In addition, this study advocates for integrating psychoeducational programmes on self-perception into school-based anti-bullying policies, which could empower adolescents to manage their negative self-evaluations and build resilience against bullying acts.

Despite these implications, the current research has some limitations that must be highlighted. First, the moderation models were tested through an online cross-sectional design. This approach examines the relationships between the research variables at a single point, allowing only directionality and causality hypotheses to be made. Future research should use a longitudinal design to provide more robust conclusions. Second, this study was conducted with a relatively small sample, which may limit the generalisability of the results. To improve the robustness of our findings, future research should confirm the moderation effect of gender in the association between hated self and bullying victimisation with a larger and more gender-balanced sample. Third, the data were collected in a single cultural and geographical context (Western-central Italian context). As the relationship between self-criticism and bullying victimisation may fluctuate in different cultural contexts, leading to various levels of bullying and cyberbullying that may differ according to gender, cross-cultural and multinational studies should be planned. Fourth, bullying was evaluated through a self-report scale, which does not allow for exploring the features of bullying deeply. Future research should use qualitative methods, like structured and semi-structured interviews, to address this issue more effectively. Finally, even though self represents a critical factor in explaining individual differences, other variables should be considered to better understand the main individual factors underpinning the risk of bullying victimisation, and consequently undermining overall well-being and quality of life, such as personality traits and cognitive styles [81,82,83].

## 5. Conclusions

In conclusion, this study provides deeper insights into the complex relationship between self-perception and bullying victimisation, highlighting the moderating role of gender. The findings indicate that gender specifically moderates the association between hated self and bullying victimisation, underscoring the need to consider gender differences in understanding victimisation dynamics. Overall, these results contribute to the growing body of research on bullying, reinforcing the importance of developing targeted prevention strategies to enhance adolescents’ well-being and resilience.

## Figures and Tables

**Table 1:** Correlation among study variables.

	1.	2.	3.	4.	5.	6.
1. Age	1					
2. Gender	−0.02	1				
3. Inadequate self	0.13 *	−0.17 **	1			
4. Hated self	0.02	−0.11 *	0.56 **	1		
5. Reassuring self	−0.11	0.24 **	−0.39 **	−0.42 **	1	
6. Bullying victimisation	−0.02	−0.09	0.26 **	0.26 **	−0.12 *	1

Note. N = 328. Gender (0 = F; 1 = M) was dummy-coded. ** *p* < 0.01 (two-tailed). * *p* < 0.05 (two-tailed).

## Data Availability

The data presented in this study are available on request from the corresponding author due to privacy reasons.
